# An App-Based Surveillance System for Undergraduate Students’ Mental Health During the COVID-19 Pandemic: Protocol for a Prospective Cohort Study

**DOI:** 10.2196/30504

**Published:** 2021-09-17

**Authors:** Chris Brogly, Michael A Bauer, Daniel J Lizotte, MacLean L Press, Arlene MacDougall, Mark Speechley, Erin Huner, Marc Mitchell, Kelly K Anderson, Eva Pila

**Affiliations:** 1 Faculty of Information and Media Studies Western University London, ON Canada; 2 Faculty of Health Sciences Western University London, ON Canada; 3 Department of Computer Science Western University London, ON Canada; 4 Department of Epidemiology and Biostatistics Schulich School of Medicine and Dentistry Western University London, ON Canada; 5 School of Kinesiology Faculty of Health Sciences Western University London, ON Canada; 6 Department of Psychiatry Schulich School of Medicine and Dentistry Western University London, ON Canada; 7 Department of Geography and Envrionment Faculty of Social Sciences Western University London, ON Canada; 8 Ivey Business School Western University London, ON Canada

**Keywords:** undergraduate, mental health, smartphone, app, COVID-19, postsecondary institutions, mobile apps, mHealth, mobile health

## Abstract

**Background:**

The COVID-19 pandemic is a public health emergency that poses challenges to the mental health of approximately 1.4 million university students in Canada. Preliminary evidence has shown that the COVID-19 pandemic had a detrimental impact on undergraduate student mental health and well-being; however, existing data are predominantly limited to cross-sectional survey-based studies. Owing to the evolving nature of the pandemic, longer-term prospective surveillance efforts are needed to better anticipate risk and protective factors during a pandemic.

**Objective:**

The overarching aim of this study is to use a mobile (primarily smartphone-based) surveillance system to identify risk and protective factors for undergraduate students’ mental health. Factors will be identified from weekly self-report data (eg, affect and living accommodation) and device sensor data (eg, physical activity and device usage) to prospectively predict self-reported mental health and service utilization.

**Methods:**

Undergraduate students at Western University (London, Ontario, Canada), will be recruited via email to complete an internet-based baseline questionnaire with the option to participate in the study on a weekly basis, using the Student Pandemic Experience (SPE) mobile app for Android/iOS. The app collects sensor samples (eg, GPS coordinates and steps) and self-reported weekly mental health and wellness surveys. Student participants can opt in to link their mobile data with campus-based administrative data capturing health service utilization. Risk and protective factors that predict mental health outcomes are expected to be estimated from (1) cross-sectional associations among students’ characteristics (eg, demographics) and key psychosocial factors (eg, affect, stress, and social connection), and behaviors (eg, physical activity and device usage) and (2) longitudinal associations between psychosocial and behavioral factors and campus-based health service utilization.

**Results:**

Data collection began November 9, 2020, and will be ongoing through to at least October 31, 2021. Retention from the baseline survey (N=427) to app sign-up was 74% (315/427), with 175-215 (55%-68%) app participants actively responding to weekly surveys. From November 9, 2020, to August 8, 2021, a total of 4851 responses to the app surveys and 25,985 sensor samples (consisting of up to 68 individual data items each; eg, GPS coordinates and steps) were collected from the 315 participants who signed up for the app.

**Conclusions:**

The results of this real-world longitudinal cohort study of undergraduate students’ mental health based on questionnaires and mobile sensor metrics is expected to show psychosocial and behavioral patterns associated with both positive and negative mental health–related states during pandemic conditions at a relatively large, public, and residential Canadian university campus. The results can be used to support decision-makers and students during the ongoing COVID-19 pandemic and similar future events. For comparable settings, new interventions (digital or otherwise) might be designed using these findings as an evidence base.

**International Registered Report Identifier (IRRID):**

DERR1-10.2196/30504

## Introduction

There are approximately 1.4 million students enrolled at Canadian universities [1], and more than 50% of students report feeling overwhelmed, lonely, anxious, and depressed during typical periods of study [2]. The presence of mental health concerns places a demand on campus mental health services, which has only escalated as students cope with the psychological effects of the COVID-19 pandemic [3,4]. Since the start of the pandemic, undergraduate students have faced abrupt campus-based restrictions, displacement from on-campus living and learning environments, increased financial uncertainty, and unprecedented shifts to web-based learning. Further, the necessary physical distancing policies have diminished opportunity for social connection, which is important for coping, particularly during a public health crisis [5]. Preliminary data on undergraduate students have shown detrimental impacts on mental health and psychological well-being [4,6-8]. For example, over 100,000 postsecondary students from across Canada participated in a web-based Statistics Canada–sponsored questionnaire about the impacts of the COVID-19 pandemic. In Spring 2020, students reported significant concern about the pandemic affecting their academic performance, the disruption of their studies, loss of work, and increased concerns about their financial stability [9,10]. As the pandemic progressed to 2021, research involving undergraduates in the United States, Canada, and China continued to show that undergraduate students were experiencing a multitude of difficulties related to their well-being (eg, financial situation and food security) and particularly their mental health (eg, depression and anxiety) as a result of the pandemic [2,3,7-14].

However, notwithstanding some published studies [15-18], the existing knowledge on undergraduate student mental health relies primarily on cross-sectional survey-based research. Owing to the rapidly evolving nature of the pandemic, public health restrictions, and the current vaccine rollout, research is needed to examine the dynamic changes in student mental health. Earlier recommendations for the psychological management of COVID-19 urged widespread surveillance efforts [19], which are critical in anticipating the forthcoming mental health needs for students on campus [20]. Following this, studies that successfully used mobile surveillance systems to examine student mental health during the pandemic are now emerging [17,18].

However, to the best of our knowledge, none of these studies have been performed at a Canadian university and linked to campus-based administrative mental health utilization data. As such, there is a unique opportunity and immediate need to use a mobile surveillance system to identify the personal and contextual risk and protective factors, which might prospectively predict mental health among Canadian undergraduate students, further contributing to the international results emerging in this area. This protocol describes a study that integrates 3 separate data sources—self-reported survey responses, mobile sensor data, and campus-based health administrative data—to identify indicators associated with student mental health. This method of data acquisition based on our designed software will aid in devising strategies to support vulnerable student groups and potentially mitigate the adverse effects of COVID-19 and other future pandemics.

The specific aims of this study are to estimate cross-sectional associations among student characteristics (eg, living accommodation, previous on-campus service utilization, and demographics such as gender/sex), key psychosocial factors (eg, affect, stress, coping, and social support), and behaviors (eg, physical activity, sleep, and device usage) during the pandemic. This study will identify factors associated with student mental health and coping responses during the pandemic. Additionally, longitudinal associations between key psychosocial (eg, affect, stress, coping, and social support) and behavioral factors (eg, physical activity, sleep, and device usage) during the pandemic and key health service utilization outcomes (eg, frequency of visits, diagnoses, and treatments) will be estimated. We anticipate that the estimation of the previously described cross-sectional and longitudinal associations will allow for the identification of indicators of mental health and coping responses during the COVID-19 pandemic for new interventions, and may facilitate a prospective prediction of mental health outcomes by using machine learning methods.

## Methods

### App Development

Members of this research group (CB, MAB, and DJL) implemented a new software suite called Ecological Momentary Assessment eXtensions 1 (EMAX1) in 2019, which consisted of a server and customizable mobile app for surveys and device sensor data collection. Previous software in this area tend to rely on uploads of large data batches. One example is StudentLife, which was used for research on university student mental health since 2014 [21] and remains in successful use as of 2021 [18]. In comparison, EMAX opts for more restrained and transaction-based data collection to manage ethical complexities around the collection of personal and potentially identifiable data on a university campus [22]. In addition to StudentLife, software comparable to EMAX has existed for some time, such as (but not limited to) Funf (2011) [23], Mobilyze (2011) [24], and Beiwe (2016) [25]. EMAX further contributes to this space as an up-to-date solution which is expected to be open-sourced/improved [22] for general smartphone-based EMA-type research.

EMAX1 was used to facilitate the development of a compact, digitized version of an existing study called Smart Healthy Campus (SHC). SHC investigated overviews of undergraduate mental health by using long internet-based questionnaires. EMAX1 was used as a cost-effective, customizable software solution to eliminate the disadvantages of using long questionnaires while adding the capability to collect a range of mobile device sensor readings and explore how they might relate to undergraduate students’ mental health profiles. The EMAX1-based “Smart Healthy Campus” app was published on Android/iOS. A publication on the preliminary SHC formative work was accepted during this protocol submission [26].

**Figure 1:**
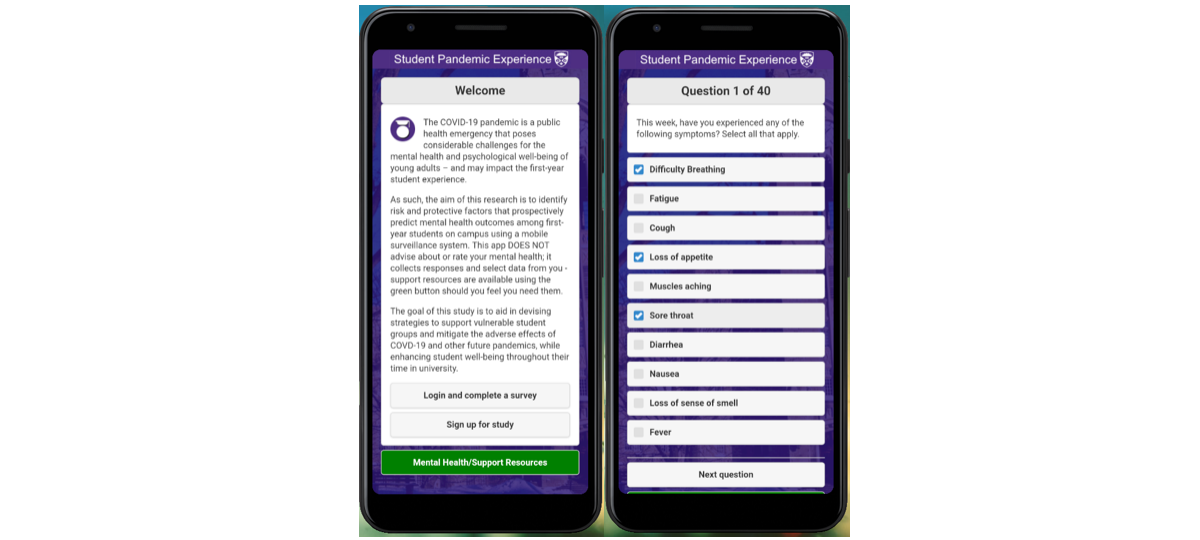
The Student Pandemic Experience (SPE) App.

For the current study the EMAX1 software was repurposed and upgraded to accommodate the pandemic context. Specifically, the EMAX2 upgrade was implemented to accommodate new surveys focusing on the pandemic, background data collection events, and additional sensor items (described in Textbox 1). As EMAX2 was developed, a new Android/iOS app based on it was branded as “Student Pandemic Experience” to match the study name, shown in Figure 1. At the same time, the protocol for Student Pandemic Experience was developed with a comprehensive baseline survey assessing sociodemographic and broad indicators of mental health, and brief weekly follow-up surveys measuring time-varying psychological constructs (eg, affect and health behavior engagement). The SPE study received Health Sciences Research Ethics Board approval from Western University in October 2020 and recruitment began in November 2020 when the iOS version of the SPE app was released. The release of the SPE Android app followed in December 2020. A separate paper to describe the EMAX2 platform in technical detail will be available for September 2021 [22].

For the SPE app, there is a maximum of 68 individual data items that may be recorded by our server when certain events occur, such as a questionnaire response event or background data collection event. Table 1 displays these, although each of the 68 data items are not listed on individual rows for brevity. Individual components of key items (such as GPS coordinates) are included as part of the 68-item total. Some items may not be sent depending on participant permissions for the SPE app. There are differences between Android and iOS, which only allowed certain items to be collected on each platform. The category “Mental Health and Support Resources panel” refers to data collection features of a panel from our EMAX2-based apps, which lists mental health–related services and organizations local to Western University (London, Ontario, Canada), with their weblinks and telephone numbers.

**Table 1:** A summary of the data collection features of the Ecological Momentary Assessment eXtensions 2nd edition–based Student Pandemic Experience iOS/Android App.

Data collected			Android		iOS
**Device and participant characteristics**					
	Western university email address	✓		✓	
	Device make/model/platform	✓		✓	
	Time spent completing the survey	✓		✓	
	Time spent with the app open	✓		✓	
	Timestamps of requests	✓		✓	
	Process names of running apps and time spent executing, idling, RAM usage, and start times	✓			
	Detecting the installation of a number of popular apps	✓		✓	
	Mindfulness minutes			✓	
	Height/weight (if recorded)			✓	
	Exercise information (if recorded)			✓	
**Physical activity and sleep behavior**					
	Step count information at various times and elevations (including floors ascended or descended)	✓		✓	
	Apple bedtime information			✓	
	System sleep time	✓		✓	
	Imprecise GPS coordinates (latitude, longitude, accuracy, heading, and speed)	✓		✓	
**Active device usage**					
	System start time	✓		✓	
	Central processing unit/individual central processing unit core idle, user, and kernel time	✓		✓	
	Available RAM/total RAM	✓		✓	
	SIM Information: call state at current time	✓		✓	
	Internet connection type (WiFi/data)	✓		✓	
	Device plugged in or not	✓		✓	
	Battery level	✓		✓	
	System up time	✓		✓	
	Air temperature, ambient light level, humidity, and air pressure	✓			
	Amount of free storage space, internal or external	✓		✓	
	Counts of photos	✓		✓	
**Mental health and support resources panel with weblinks and telephone numbers**					
	Time spent with the panel open	✓		✓	
	Which resource links or phone numbers were selected (records if participants tapped phone numbers or weblinks)	✓		✓	
**Social activity**					
	Total contact counts	✓		✓	
	Family-related contact counts	✓		✓	
	Friend-related contact counts	✓		✓	
	Calendar events daily	✓		✓	
	Calendar events weekly	✓		✓	

### Study Design

SPE is a longitudinal survey–based prospective cohort study, with data collection based on the purpose-built SPE mobile app, which relies on established smartphone personal sensing capabilities [27]. It is capable of weekly repeat measures, ideally suited to studying time-varying phenomena [28]. The study does share similarities with previous EMA-based smartphone studies [16,21], although this work will have only a weekly sample rate of self-report surveys, in addition to background sensor data that are collected at multiple points daily.

### Setting

The setting is Western University from November 9, 2020, until October 31, 2021. Funds permitting, the study may extend into the Fall 2021 semester, but the study is not expected to continue to 2022. Most participants stated they were living on or near Western University’s main campus during the Fall 2020/Winter 2021 semesters; others may be located out of town simply owing to living arrangements. As the baseline survey is internet-based, and additional participation is facilitated through the SPE app, participants may contribute to the study from any location. At the time of writing, some were participating in various countries outside Canada.

Throughout the pandemic, significant outbreak events occurred in residence buildings and in predominantly student living areas in London (Ontario, Canada), similar to other universities [29]. Considering that predominantly student living areas can be COVID-19 hotspots and that restrictive measures changed, we believe this justifies the weekly repeat measures and hourly sensor data collections for this investigation of an overview of undergraduate mental health. Additionally, provincial/university restrictive measures are expected to influence participant location. For instance, Western University’s residences were not fully open again in 2021, until February, as Western University delayed opening its residences owing to the provincial lockdown.

### Recruitment and Consent

Through campus-wide emailing systems, all Western University undergraduate students were invited via mass email to complete a web-based baseline survey. To complete the baseline survey, a participant had to view and accept the letter of information and consent for the study. After completing the baseline survey, participants received another email with information on the SPE app and Snapchat and quick response codes to download. If participants chose to continue with weekly participation through the SPE app, they were required to view the letter of information again during the sign-up process in the app and were also able to consent for linkage with their records from administrative sources on campus (health and psychological services).

### Inclusion and Exclusion Criteria

The initial inclusion criterion was that a participant would be a first-year undergraduate student at Western University; to increase enrollment, in January 2021, this criterion was relaxed to include any undergraduate students. Exclusion criteria were an inability to provide written informed consent or to complete surveys or forms owing to language or cognitive difficulties; students enrolled in graduate or professional programs; or students enrolled and actively participating in the SHC study, which employed a similar version of the software and was initiated prior to the pandemic.

### Participant Compensation

Participants will be remunerated for time spent completing surveys. Participants who submitted the internet-based baseline surveys will be compensated with a Can $10 Amazon e-gift card. Participants who complete at least 3 of 4 weekly surveys each month will be compensated with a Can $10 Amazon e-gift card per month. This compensation structure was intended to optimize baseline recruitment and promote participant retention over time.

### Baseline Survey

The SPE baseline survey is composed primarily of existing psychometrically tested instruments that demonstrate validity evidence, and takes approximately 30 minutes to complete. It is delivered on the internet through the Qualtrics XM platform. Participants were asked about sociodemographic factors, current living arrangement, physical and mental health history, social support, and COVID-19–related risks. Participants may skip any questions they prefer not to answer. The composition of the baseline survey is outlined below in Textbox 1. Many COVID-19 questions were adapted from The Healthy Minds Network + American College Health Association COVID HMS Survey Items Spring 2020 [30].

**Baseline survey items and questionnaires.**

**Sociodemographics**

- Age
- Sex & gender
- Ethnicity
- Sexual minority status
- Relationship status
- Socioeconomic status

**Campus/student data**

- Faculty name
- Degree (based on faculty)
- Program year
- Place of residence (on campus in residence, off campus in London, or off campus outside London)
- Classes enrolled in
- Time spent web-based vs in-person learning

**Diagnosed mental illness**

- Lifetime diagnosis

**COVID-19 assessment and history**

- Previous diagnosis of COVID-19
- If yes, symptom severity
- If yes, hospitalization
- Engaging in recommended hygiene practices
- COVID-19–related discrimination

**Physical activity**

- Leisure Time Physical Activity Questionnaire [31]

**Perceived social connection**

- Social Connectedness Scale 8-items [32]

**Depressive symptoms**

- Patient Health Questionnaire 9 items [33]

**Anxiety symptoms**

- Generalized Anxiety Scale, 7-item [34]

**Flourishing**

- Flourishing Scale, 8-item [35]

**Resilience**

- Brief Resilience Scale, 6-item [36]

**Self-compassion**

- Self-Compassion Scale Short Version [37]

**Body appreciation**

- Body Appreciation Scale-2 [38]

**Self-worth**

- Contingencies of Self-Worth Scale [39]

**Weight bias**

- Weight Bias Internalization Scale [40]

**Self-objectification**

- Self-Objectification Beliefs & Behaviours Scale [41]

### SPE App and Weekly Surveys

The SPE app facilitates the distribution of the weekly surveys should participants choose to continue with the study past the baseline survey. An app survey takes approximately 5 minutes to complete each week. Push notifications are sent to participants weekly to remind them to complete a brief report of their psychosocial states and behaviors over the past week. Similar to the baseline survey, they are primarily based on existing validated instruments with some new items for COVID-19. COVID-19–related questions include 2 questions for reporting on COVID-19 symptoms. A notification is set up on the app, so that, if participants report in their questionnaire responses experiencing at least 1 important COVID-19 symptom (such as difficulty breathing, fatigue, cough, loss of appetite, muscles aching, sore throat, diarrhea, nausea, loss of sense of smell, or fever), they are prompted to visit the Ontario government’s COVID-19 self-assessment website [42] and be provided with recommendations. Textbox 2 outlines the biweekly assessment items.

The SPE app supports other features in addition to administering the questionnaire. Background data collection occurs every 1, 2, or 3 hours depending on the participant setting. When the app is closed, background data collection stops. The app also includes a “Mental Health Resources” panel that offers various support services relevant to Western University’s undergraduate students. If a participant uses this panel, actions including clicking on weblinks or telephone numbers to the services are recorded. The app is designed to collect data from device sensors, such as GPS coordinates, physical steps, and other device and use characteristics (as shown in Table 1). When a participant starts a questionnaire, completes a questionnaire, uses the mental health resource panel, or when the app triggers a background data collection event, based on the participant setting, data are collected through the app. All data are sent over HTTPS and are further encrypted when stored by the app server; GPS coordinates are first encrypted on the device with RSA before being sent over HTTPS.

**Biweekly assessment items.**

**Mental health symptoms**

- Depression Anxiety and Stress Scale [43]

**Self-compassion**

- 6-item weekly self-compassion items [44]

**COVID-19 monitoring**

- Symptom monitoring (adapted from Health Canada)
- Pandemic worry
- Impact of pandemic on academics
- Impact of pandemic on mental well-being
- Impact of pandemic on first-year experience

**Substance use**

- Binge drinking in past 2 weeks
- Use of recreational substances in past 2 weeks

**Affect**

- Positive and Negative Affective Schedule [45]

**Body emotions**

- 4-item body self-conscious emotions [46]

**COVID-19 monitoring**

- Symptom monitoring (adapted from Health Canada)
- Impact of pandemic on first-year experience
- Pandemic worry
- Impact of pandemic on academics
- Impact of pandemic on mental well-being

**Dietary restraint**

- Restrict food intake to influence shape/weight in past 2 weeks [47]

**Physical activity**

- Moderate-to-vigorous physical activity in past 2 weeks
- Strength training in past 2 weeks
- Primary reason for exercising

### Data Preparation and Analysis

SPSS, R, and Python will be used for data cleaning and analysis of items described in Tables 1 and 2, as required. Additional tools may also be used. The data infrastructure we develop for subsequent publications is expected to support a wide range of analyses, ranging from confirmatory (eg, studying the association between COVID-19 perceptions and mental health utilization) to exploratory (eg, identifying clusters of students with similar psychological profiles). The identification of student clusters based on the psychological profile (derived from questionnaire responses and mobile sensor data) is expected to reveal various mental health–related states, which may range from positive to negative, and predict campus-based mental health utilization. Taken together, this information might then be used to identify students at higher risk and inform decision-making or build evidence-based interventions (digital or otherwise) that may target at-risk individuals, and broadly promote indices of positive mental health and reduce indices of negative mental health.

Initial analyses will use the cross-sectional data to assess univariate and multivariable associations between psychosocial and behavioral factors and outcomes, including health and psychological service utilization. Following this, the longitudinal data will be used to assess these associations and their change over time by applying repeated measures techniques. Statistical comparisons might include (but will not be limited to) *t* tests, analysis of variance, the Mann–Whitney *U* test, Kruskal–Wallis test, Pearson/Spearman correlation analyses, and linear and multiple regression analyses. This will be followed by exploratory analyses using machine learning methods to identify latent structure in the data and to develop predictive models for mental health outcomes. Additionally, a log of key events related to the COVID-19 pandemic will be compiled during data collection. The log may include items such as on-campus outbreaks and vaccine developments. Potential associations between these events and study data will be explored during analysis. As this is an exploratory study examining a constantly evolving health and social phenomenon, additional research questions and analyses are expected to arise as the study progresses.

**Table 2:** Data collection summary from November 9, 2020, to August 8, 2021.

Data	Value, n
Participants	315
Survey responses	4851
Sensor samples	25,985

## Results

### Participation and Data Collection Summary

After 2 months of first year–only recruitment from November 9 to December 31, 2020, a total of 87 first year students were actively participating. To obtain a larger participant group, we amended the study in January 2021 to allow for recruitment of all undergraduate students; a total of 427 completed the baseline survey. In total, 315/427 (74%) students signed up to use the SPE app and 266/315 (84%) completed at least 1 weekly app survey. Recruitment efforts ended in February 2021. From mid-February to April 2021, weekly app usage was in a steady range of 175-215 participants. Regular participation declined over the summer term from May to August 2021, and by August 2021 there were approximately 95 participants still responding to surveys. Data collection, at the time of this writing, was conducted from November 9 to August 8, 2021, and data collection was planned to conclude October 31, 2021. Data collection efforts are summarized below in Table 2. Each participant completed at least 1 survey. Each “sensor sample” consists of all the items in Table 1, although certain values for items may be null owing to participant telephone permissions (they may deny GPS access, for instance) or data simply being unavailable depending on the situation or smartphone model.

The analysis of the survey responses (representative of mental health–related states) and sensor sample data (representative of associated lifestyle/behavior) is not provided in this protocol paper and will be the main component of a subsequent publication.

### Demographics

Below, Table 3 outlines key aspects of participant demographics, and Table 4 shows key participant experiences with COVID-19. These results were taken from the SPE internet-based baseline survey. Owing to their length, some data that were collected were omitted from these tables. The full versions of the tables are provided in Multimedia Appendix 1.

**Table 3:** Participant demographics at baseline. Additionally, as questions can be skipped, sometimes the cells for each item may not add to 100%.

Data			Completed baseline survey and at least 1 app survey (n=266), %		Completed baseline survey only (n=161), %
**What is your gender identity?**					
	Man	22.9		24.9	
	Woman	75.9		74.5	
	Trans man	≤5^a^		≤5	
	Trans woman	≤5		≤5	
	Gender queer/gender nonconforming	≤5		≤5	
**Enrollment status**					
	Full-time	75.2		80.75	
	Part-time	≤5		≤5	
	Missing	20		19.3	
**Enrollment year**					
	First year	22.2		23.6	
	Second year	18.8		11.8	
	Third year	17.7		19.3	
	Fourth year	14.6		23.6	
	≥Fifth year	≤5		≤5	
**Device type**					
	Android	14.7		22.4	
	iOS	85.3		77.6	
**How would you characterize your relationship status?**					
	Single	56.8		55.9	
	In a relationship	42.1		41	
	Married, domestic partnership, engaged	≤5		≤5	
	Missing	≤5		≤5	
**Housing situation**					
	On or off campus non-university housing in London, Ontario	51.5		48.4	
	On-campus housing	24.4		27.3	
	Outside of London, Ontario	21.1		21.1	
	Other	≤5		≤5	
**Proportion of classes enrolled in primarily web-based learning?**					
	All classes	81.9		73.7	
	Some classes	28.1		26.3	
**Diagnosis for mental health condition**					
	None	59.4		58.4	
	Do not know	≤5		≤5	
	Anxiety	9		6	
	Bipolar disorder	≤5		≤5	
	Depression	20		20.5	
	Eating disorder	≤5		≤5	
	Neurodevelopmental disorder (eg, attention-deficit/hyperactivity disorder)	≤5		≤5	
	Obsessive-compulsive or related disorder (eg, body dysmorphia)	≤5		≤5	
	Substance use disorder	≤5		≤5	
	Trauma and stressor related disorders (eg, posttraumatic stress disorder)	≤5		≤5	

^a^For privacy reasons, “≤5%” is used in some cells.

**Table 4:** Experience with COVID-19 at baseline. Additionally, as questions can be skipped, sometimes the cells for each item may not add to 100%.

Data			Completed baseline survey and at least 1 app survey (n=266), %		Completed baseline survey only (n=161), %
**Have you had COVID-19?**					
	Yes (confirmed by a test)	≤5^a^		≤5	
	Probably (eg, a health care provider told me that I likely had COVID-19, but it was not confirmed by a test)	≤5		≤5	
	Maybe (eg, I have had symptoms consistent with COVID-19, but it was not confirmed through a test)	7.9		7.5	
	No (no symptoms or other reason to think I have had it)	89.5		90	
**How severe were any of the symptoms of COVID-19?^b^**					
	Severe (eg, difficulty breathing or speaking, low blood pressure, and a high fever of 103°F [39.4°C] or higher)	≤5		≤5	
	Moderate (eg, some shortness of breath, cough, a fever of 100.4°F [38°C] or higher, or mild [eg, cold-like symptoms])	8.3		6.2	
	No symptoms (asymptomatic)	≤5		≤5	
**How likely do you think you will get COVID-19?^c^**					
	Very likely	≤5		≤5	
	Likely	≤5		6.2	
	Somewhat likely	47		42.9	
	Not at all likely	36.8		41%	
**To what extent have you been following recommendations for hygiene practices (frequent hand-washing; avoiding touching your eyes, nose, and mouth; and disinfecting surfaces)?**					
	Not at all following recommendations	≤5%		≤5%	
	Not closely following recommendations	≤5%		6.2%	
	Somewhat closely following recommendations	46.2%		46.6%	
	Very closely following recommendations	49.2%		47.2	
**To what extent have you been following recommendations for social/physical distancing (maintaining a 6-foot distance between yourself and others in public, avoiding large gatherings, and avoiding nonessential trips outside of home)?**					
	Not at all following recommendations	≤5		≤5	
	Not closely following recommendations	≤5		6.8	
	Somewhat closely following recommendations	46.2		50.9	
	Very closely following recommendations	49.2		41	

^a^For privacy reasons, ≤5% is used in some cells.

^b^Only asked to students who reported having had COVID-19.

^c^May include students that previously disclosed infection.

## Discussion

### Expected Outcome

This novel and integrated surveillance paradigm will support action to address student mental health during an unprecedented public health crisis. The outcomes of this surveillance study will provide postsecondary institutions with critical data to support evidence-informed planning and decision-making after a pandemic, as well as ongoing resourcing of student mental health supports. Further, the SPE app will be used as a model for the development of other institution-specific tools that combine student-level data, campus-wide administrative data, and longitudinal self-reported data. By examining associations between (1) student characteristics and key psychosocial and behavioral factors and (2) how these factors predict health service utilization, this study will be essential to building capacity at institutions for the early estimation of student mental health concerns, as well as informing the creation of agile and responsive institutional policies during a public health crisis.

### Impact

To the best of our knowledge, there are no comparable studies conducted on student mental health at any Canadian institution during a pandemic such as COVID-19. A study from a US Ivy League university on student mental health during the pandemic has been conducted with similar data collection techniques by using a platform that pre-dates our EMAX software [17,18]. Although aspects of the university experience in Canada might be comparable to those at institutions from other studies on student mental health, it still is a nuanced experience that may not be fully represented by other studies in the area. Further, this study will be the first to integrate administrative campus-based mental health utilization data with longitudinal self-report and sensor data on mental health. Therefore, this research is expected to be of particular interest to post-secondary institution stakeholders and policy makers in Canada. In particular, this study has the potential to inform other postsecondary institutions that are considered a residential campus, and thus comparable to Western University. Our results are also expected to provide a foundation on which new evidence-based interventions in lifestyle (digital or otherwise) might be designed to improve student mental health at Canadian institutions, given our combination of mental health–related assessment via questionnaires with its associated sensor data collected from cellphones.

### Limitations

There are some limitations to the study. For instance, the data collected during the end of 2020 were limited to first-year students only, and were expanded to all undergraduate students at the beginning of 2021. Additionally, despite our EMAX-based SPE app being able to collect up to 68 values from participants, in practice we will receive less than that number since many items were optional with cellphone permissions. Another potential limitation is that participants might not have always had their cellphones with them during lockdowns, which might impact our results.

### Conclusions

Given the absence of longitudinal data on student lifestyle and indicators of mental health during a pandemic in Canada, this study is expected to produce a unique assessment of undergraduates with our mobile surveillance system, which has captured aspects of day-to-day life combined with a significant number of weekly responses. Analysis of these data is expected to yield various mental health-related states associated with academic and pandemic-related events. Our results are expected to be of use for the design of interventions to improve student mental health during a pandemic and to institution stakeholders who may benefit from the results for policy and decision-making.

## Appendix

Multimedia Appendix 1 Full baseline survey demographics - extended Table 5 and 6.

## Abbreviations

EMA: Ecological Momentary Assessment
EMAX1: Ecological Momentary Assessment eXtensions, 1st edition
EMAX2: Ecological Momentary Assessment eXtensions, 2nd edition
SHC: Smart Healthy Campus
SPE: Student Pandemic Experience

## References

[1] Facts and stats. Universities Canada.

[2] American College Health Association (2007). American College Health Association National College Health Assessment Spring 2006 Reference Group data report (abridged). J Am Coll Health.

[3] Cao W, Fang Z, Hou G, Han M, Xu X, Dong J, Zheng J (2020). The psychological impact of the COVID-19 epidemic on college students in China. Psychiatry Res.

[4] Wang X, Hegde S, Son C, Keller B, Smith A, Sasangohar F (2020). Investigating Mental Health of US College Students During the COVID-19 Pandemic: Cross-Sectional Survey Study. J Med Internet Res.

[5] Yin-xia Bai, Gegentuya H, Hu Liu, Zhen-Hua Wang, Wen-Rui Wa, Zhi-Gang NG (2005). Correlation Between Psychological Changes of The Community Crowd and The Social Support in Grave Public Health Event. Nei Moivgol Med J.

[6] Ma Z, Zhao J, Li Y, Chen D, Wang T, Zhang Z, Chen Z, Yu Q, Jiang J, Fan F, Liu X (2020). Mental health problems and correlates among 746 217 college students during the coronavirus disease 2019 outbreak in China. Epidemiol Psychiatr Sci.

[7] Zhai Y, Du X (2020). Addressing collegiate mental health amid COVID-19 pandemic. Psychiatry Res.

[8] Zhai Y, Du X (2020). Mental health care for international Chinese students affected by the COVID-19 outbreak. Lancet Psychiatry.

[9] Doreleyers A, Knighton T (2020). COVID-19 Pandemic: Academic Impacts on Postsecondary Students in Canada. Statistics Canada.

[10] (2020). COVID-19 Pandemic: financial impacts on postsecondary students in Canada. Statistics Canada.

[11] Mialki K, House LA, Mathews AE, Shelnutt KP (2021). Covid-19 and College Students: Food Security Status before and after the Onset of a Pandemic. Nutrients.

[12] Son C, Hegde S, Smith A, Wang X, Sasangohar F (2020). Effects of COVID-19 on College Students' Mental Health in the United States: Interview Survey Study. J Med Internet Res.

[13] Tasso AF, Hisli Sahin N, San Roman GJ (2021). COVID-19 disruption on college students: Academic and socioemotional implications. Psychol Trauma.

[14] Molock SD, Parchem B (2021). The impact of COVID-19 on college students from communities of color. J Am Coll Health.

[15] Fried E, Papanikolaou F, Epskamp S (2020). Mental Health and Social Contact During the COVID-19 Pandemic: An Ecological Momentary Assessment Study. PsyArXiv. Preprint published online April 24, 2020.

[16] Kleiman EM, Yeager AL, Grove JL, Kellerman JK, Kim JS (2020). Real-time Mental Health Impact of the COVID-19 Pandemic on College Students: Ecological Momentary Assessment Study. JMIR Ment Health.

[17] Huckins JF, daSilva AW, Wang W, Hedlund E, Rogers C, Nepal SK, Wu J, Obuchi M, Murphy EI, Meyer ML, Wagner DD, Holtzheimer PE, Campbell AT (2020). Mental Health and Behavior of College Students During the Early Phases of the COVID-19 Pandemic: Longitudinal Smartphone and Ecological Momentary Assessment Study. J Med Internet Res.

[18] Mack DL, DaSilva AW, Rogers C, Hedlund E, Murphy EI, Vojdanovski V, Plomp J, Wang W, Nepal SK, Holtzheimer PE, Wagner DD, Jacobson NC, Meyer ML, Campbell AT, Huckins JF (2021). Mental Health and Behavior of College Students During the COVID-19 Pandemic: Longitudinal Mobile Smartphone and Ecological Momentary Assessment Study, Part II. J Med Internet Res.

[19] Qiu J, Shen B, Zhao M, Wang Z, Xie B, Xu Y (2020). A nationwide survey of psychological distress among Chinese people in the COVID-19 epidemic: implications and policy recommendations. Gen Psychiatr.

[20] Mei SL, Yu JX, He BW, Li JY (2011). Psychological investigation of university students in a university in Jilin Province. Med Soc (Berkeley).

[21] Wang R, Chen F, Chen Z, Li T, Harari G, Tignor S, Zhou X, Ben-Zeev D, Campbell AT (2014). StudentLife: assessing mental health, academic performance and behavioral trends of college students using smartphones.

[22] Brogly C, Lizotte D, Bauer M (2021). Ecological Momentary Assessment eXtensions 3 (EMAX3) Proposal: An App for EMA-Type Research.

[23] Aharony N, Pan W, Ip C, Khayal I, Pentland A (2011). Social fMRI: Investigating and shaping social mechanisms in the real world. Pervasive Mob Comput.

[24] Burns MN, Begale M, Duffecy J, Gergle D, Karr CJ, Giangrande E, Mohr DC (2011). Harnessing context sensing to develop a mobile intervention for depression. J Med Internet Res.

[25] Torous J, Kiang MV, Lorme J, Onnela J (2016). New Tools for New Research in Psychiatry: A Scalable and Customizable Platform to Empower Data Driven Smartphone Research. JMIR Ment Health.

[26] Brogly C, Shoemaker JK, Lizotte DJ, Kueper JK, Bauer MA (2021). Smart Healthy Campus: A mobile application to identify lifestyle indicators related to undergraduate mental health. JMIR Form Res.

[27] Mohr DC, Zhang M, Schueller SM (2017). Personal Sensing: Understanding Mental Health Using Ubiquitous Sensors and Machine Learning. Annu Rev Clin Psychol.

[28] Almeida DM (2016). Resilience and Vulnerability to Daily Stressors Assessed via Diary Methods. Curr Dir Psychol Sci.

[29] Fox MD, Bailey DC, Seamon MD, Miranda ML (2021). Response to a COVID-19 Outbreak on a University Campus - Indiana, August 2020. MMWR Morb Mortal Wkly Rep.

[30] The Healthy Minds Network, American College Health Association The impact of COVID-19 on college student well-being. American College Health Association.

[31] Godin G (2011). The Godin-Shephard Leisure-Time Physical Activity Questionnaire. Heal Fit J Canada.

[32] Lee RM, Robbins SB (1995). Measuring belongingness: The Social Connectedness and the Social Assurance scales. J Couns Psychol.

[33] Kroenke K, Spitzer RL, Williams JBW (2001). The PHQ-9: validity of a brief depression severity measure. J Gen Intern Med.

[34] Spitzer RL, Kroenke K, Williams JBW, Löwe B (2006). A brief measure for assessing generalized anxiety disorder: the GAD-7. Arch Intern Med.

[35] Diener E, Wirtz D, Tov W, Kim-Prieto C, Choi D, Oishi S, Biswas-Diener R (2009). New Well-being Measures: Short Scales to Assess Flourishing and Positive and Negative Feelings. Soc Indic Res.

[36] Smith BW, Dalen J, Wiggins K, Tooley E, Christopher P, Bernard J (2008). The brief resilience scale: assessing the ability to bounce back. Int J Behav Med.

[37] Raes F, Pommier E, Neff KD, Van Gucht D (2011). Construction and factorial validation of a short form of the Self-Compassion Scale. Clin Psychol Psychother.

[38] Tylka TL, Wood-Barcalow NL (2015). The Body Appreciation Scale-2: item refinement and psychometric evaluation. Body Image.

[39] Crocker J, Wolfe CT (2001). Contingencies of self-worth. Psychol Rev.

[40] Pearl RL, Puhl RM (2014). Measuring internalized weight attitudes across body weight categories: validation of the modified weight bias internalization scale. Body Image.

[41] Lindner D, Tantleff-Dunn S (2017). The Development and Psychometric Evaluation of the Self-Objectification Beliefs and Behaviors Scale. Psychol Women Q.

[42] COVID-19 self-assessment. Ontario.

[43] Lovibond P, Lovibond SH (1995). The structure of negative emotional states: comparison of the Depression Anxiety Stress Scales (DASS) with the Beck Depression and Anxiety Inventories. Behav Res Ther.

[44] NEFF KD (2003). The Development and Validation of a Scale to Measure Self-Compassion. Self Identity.

[45] Watson D, Clark LA, Tellegen A (1988). Development and validation of brief measures of positive and negative affect: the PANAS scales. J Pers Soc Psychol.

[46] Tracy JL, Robins RW (2004). TARGET ARTICLE: "Putting the Self Into Self-Conscious Emotions: A Theoretical Model". Psychol Inq.

[47] Luce KH, Crowther JH (1999). The reliability of the Eating Disorder Examination-Self-Report Questionnaire Version (EDE-Q). Int J Eat Disord.

