# Supplementary material for: An App-Based Surveillance System for Undergraduate Students’ Mental Health During the COVID-19 Pandemic: Protocol for a Prospective Cohort Study
Source: JMIR Res Protoc. 2021 Sep 17;10(9):e30504. doi: 10.2196/30504 (PMC8451963; doi:10.2196/30504)
Supplement: Multimedia Appendix 1 [file resprot_v10i9e30504_app1.docx]

**Multimedia Appendix 1.** Full baseline survey demographics - extended Table 5 and 6.

Full Table 5: Participant demographics at baseline. For privacy reasons, <= 5% is used in some cells. Additionally, as questions can be skipped, sometimes the cells for each item may not add to 100%. Note that, due to length, this is a reduced version of the full table, which can be found as a multimedia appendix.

|  |  | **Completed baseline survey and at least one app survey**  **(N=266)** | **Completed baseline survey only (N=161)** |
| --- | --- | --- | --- |
| **How old are you (in years)?** |  |  |  |
|  | 17 and below | <= 5% | <= 5% |
|  | 18 | 32.7% | 29.1% |
|  | 19 | 17.3% | 13.6% |
|  | 20 | 15.8% | 13% |
|  | 21 | 11.3% | 24.9% |
|  | 22 | 9.4% | <= 5% |
|  | 23+ | <= 5% | <= 5% |
|  | Missing | 8.6% | 8% |
|  | Mean (SD) | 19.5 (1.980) | 19.770 (2.499) |
| **What was your sex at birth?** |  |  |  |
|  | Female | 76.7% | 75.2% |
|  | Male | 22.9% | 24.9% |
|  | Intersex | <= 5% | <= 5% |
| **What is your gender identity?** |  |  |  |
|  | Man | 22.9% | 24.9% |
|  | Woman | 75.9% | 74.5% |
|  | Trans Man | <= 5% | <= 5% |
|  | Trans Woman | <= 5% | <= 5% |
|  | Gender queer/gender nonconforming | <= 5% | <= 5% |
| **How would you describe your sexual orientation?** |  |  |  |
|  | Bisexual | 8.3% | 11.2% |
|  | Gay | <= 5% | <= 5% |
|  | Heterosexual | 83.5% | 83.2% |
|  | Lesbian | <= 5% | <= 5% |
|  | Queer | <= 5% | <= 5% |
|  | Questioning | <= 5% | <= 5% |
| **How would you describe your ethnic background?** |  |  |  |
|  | Indigenous (First Nations, Métis, and Inuit) | <= 5% | <= 5% |
|  | Black | <= 5% | <= 5% |
|  | Chinese | 22.6% | 25.5% |
|  | Filipino/a/x | <= 5% | <= 5% |
|  | Japanese | <= 5% | <= 5% |
|  | Korean | <= 5% | <= 5% |
|  | Latino/a/x | <= 5% | <= 5% |
|  | South Asian | 12.4% | 12.4% |
|  | South-East Asian | <= 5% | <= 5% |
|  | West Asian/Middle East | <= 5% | <= 5% |
|  | White/Caucasian | 42.1% | 36% |
|  | Unknown | <= 5% | <= 5% |
| **International student?** |  |  |  |
|  | Yes | <= 5% | <= 5% |
|  | No | 96.2% | 95.7% |
| **Enrollment status** |  |  |  |
|  | Full-time | 75.2% | 80.75% |
|  | Part-time | <=5% | <= 5% |
|  | Missing | 20% | 19.3% |
| **Enrollment year** |  |  |  |
|  | First year | 22.2% | 23.6% |
|  | Second year | 18.8% | 11.8% |
|  | Third year | 17.7% | 19.3% |
|  | Fourth year | 14.6% | 23.6% |
|  | Fifth year+ | <= 5% | <= 5% |
| **Device type** |  |  |  |
|  | Android | 14.7% | 22.4% |
|  | iOS | 85.3% | 77.6% |
|  |  |  |  |
| **How would you characterize your relationship status?** |  |  |  |
|  | Single | 56.8% | 55.9% |
|  | In a relationship | 42.1% | 41% |
|  | Married, domestic partnership, engaged | <= 5% | <= 5% |
|  | Missing | <= 5% | <= 5% |
| **Financial situation** |  |  |  |
|  | Always stressful | 6% | 7.5% |
|  | Often stressful | 9.4% | 20.5% |
|  | Sometimes stressful | 41% | 32.9% |
|  | Rarely stressful | 26.7% | 27.3% |
|  | Never stressful | 9.4% | 11.8% |
| **Housing situation** |  |  |  |
|  | On or off campus non-university housing in London, Ontario | 51.5% | 48.4% |
|  | On-campus housing | 24.4% | 27.3% |
|  | Outside of London, Ontario | 21.1% | 21.1% |
|  | Other | <= 5% | <= 5% |
| **Primary Faculty** |  |  |  |
|  | Arts and Humanities | <= 5% | 5.8% |
|  | Management and Organizational Sciences | <= 5% | <= 5% |
|  | Information and Media Studies | <= 5% | <= 5% |
|  | Engineering | 6.8% | 7.1% |
|  | Science | 28.2% | 29.5% |
|  | Health Sciences | 23.7% | 17.9% |
|  | Medical Sciences | <= 5% | <= 5% |
|  | Nursing | <= 5% | <= 5% |
|  | Business Administration | <= 5% | 5.8% |
|  | Social Science | 22.9% | 22.4% |
|  | Music | <= 5% | <= 5% |
|  | Other | <= 5% | <= 5% |
| **Proportion of classes enrolled in primarily virtual learning?** |  |  |  |
|  | All classes | 81.9% | 73.7% |
|  | Some classes | 28.1% | 26.3% |
| **Diagnosis for mental health condition** |  |  |  |
|  | None | 59.4% | 58.4% |
|  | Don’t know | <= 5% | <= 5% |
|  | Anxiety | 9% | 6% |
|  | Bipolar disorder | <= 5% | <= 5% |
|  | Depression | 20% | 20.5% |
|  | Eating disorder | <= 5% | <= 5% |
|  | Neurodevelopmental disorder (e.g. ADHD) | <= 5% | <= 5% |
|  | Obsessive-compulsive or related disorder (e.g. Body dysmorphia) | <= 5% | <= 5% |
|  | Substance use disorder | <= 5% | <= 5% |
|  | Trauma and stressor related disorders (e.g. PTSD) | <= 5% | <= 5% |
| **What kind of exercise do you typically do during the week?** |  |  |  |
|  | Light intensity Exercise (i.e., minimal effort) | 34% | 37.3% |
|  | Moderate intensity Exercise (i.e. not exhausting) | 29% | 23% |
|  | Strenuous intensity Exercise (i.e., Heart Beats Rapidly) | 37% | 36.7% |
| **How often do you drink alcohol?** |  |  |  |
|  | 4 or more times a week | <= 5% | <= 5% |
|  | 2-3 times a week | 11.7% | 18% |
|  | 2-4 times a month | 33.8% | 30.4% |
|  | Monthly or less | 33.8% | 29.8% |
|  | Never | 17.7% | 18.6% |
| **In the last 12 months, have you used drugs other than those required?** |  |  |  |
|  | Yes | 43.1% | 50.3% |
|  | No | 56.9% | 49.1% |

Full Table 6: Experience with COVID-19 at baseline. For privacy reasons, <= 5% is used in some cells. Additionally, as questions can be skipped, sometimes the cells for each item may not add to 100%. Note that, due to length, this is a reduced version of the full table, which can be found as a multimedia appendix.

|  |  | **Completed baseline survey and at least one app survey**  **(N=266)** | **Completed baseline survey only (N=161)** |
| --- | --- | --- | --- |
| **Have you had COVID-19?** |  |  |  |
|  | Yes (confirmed by a test) | <= 5% | <= 5% |
|  | Probably (e.g., a healthcare provider told me that I likely had COVID-19, but it was not confirmed by a test) | <= 5% | <= 5% |
|  | Maybe (e.g., I have had symptoms consistent with COVID-19, but it was not confirmed by a test) | 7.9% | 7.5% |
|  | No (no symptoms or other reason to think I have had it) | 89.5% | 90% |
| **How severe were any of the symptoms from COVID-19? *Note. Only asked to students who reported having had COVID-19*** |  |  |  |
|  | Severe (e.g., difficulty breathing or speaking, low blood pressure, high fever of 103 F (39.4 C or higher) | <= 5% | <= 5% |
|  | Moderate (e.g., some shortness of breath, cough, fever of 100.4 F (38 C) or higher) Mild (e.g., cold-like symptoms) | 8.3% | 6.2% |
|  | No symptoms (asymptomatic) | <= 5% | <= 5% |
| **Were you hospitalized because of COVID-19? *Note. Only asked to students who reported having had COVID-19*** |  |  |  |
|  | No | <= 5% | 9.3% |
| **How likely do you think you will get COVID-19? *Note. may include students that previously disclosed infection*** |  |  |  |
|  | Very likely | <= 5% | <= 5% |
|  | Likely | <= 5% | 6.2% |
|  | Somewhat likely | 47% | 42.9% |
|  | Not at all likely | 36.8% | 41% |
| **To what extent have you been following recommendations for hygiene practices (frequent hand washing; avoiding touching your eyes, nose, and mouth; and disinfecting surfaces)?** |  |  |  |
|  | Not at all following recommendations | <= 5% | <= 5% |
|  | Not closely following recommendations | <= 5% | 6.2% |
|  | Somewhat closely following recommendations | 46.2% | 46.6% |
|  | Very closely following recommendations | 49.2% | 47.2% |
| **To what extent have you been following recommendations for social/physical distancing (keeping a six-foot distance between yourself and others in public, avoiding large gatherings, and avoiding non-essential trips outside your home)?** |  |  |  |
|  | Not at all following recommendations | <= 5% | <= 5% |
|  | Not closely following recommendations | <= 5% | 6.8% |
|  | Somewhat closely following recommendations | 46.2% | 50.9% |
|  | Very closely following recommendations | 49.2% | 41% |
| **Have you experienced any discriminatory or hostile behavior due to your race/ethnicity (or what someone thought was your race/ethnicity)?** |  |  |  |
|  | No | 86.8% | 82.6% |
|  | Yes | 12% | 17.4% |
| **Have you witnessed (online exchanges or in-person) any discriminatory or hostile behavior or exchanges towards others due to their race/ethnicity (or what someone thought was their race/ethnicity)?** |  |  |  |
|  | No | 38.3% | 42.9% |
|  | Yes | 60.9% | 57.1% |
| **Have you experienced any**  **discriminatory or hostile behavior due to your weight?** |  |  |  |
|  | No | 91% | 89.4% |
|  | Yes | 8.3% | 10.6% |
